# Mechanical Power during Veno-Venous Extracorporeal Membrane Oxygenation Initiation: A Pilot-Study

**DOI:** 10.3390/membranes11010030

**Published:** 2021-01-02

**Authors:** Mirko Belliato, Francesco Epis, Luca Cremascoli, Fiorenza Ferrari, Maria Giovanna Quattrone, Christoph Fisser, Maximilian Valentin Malfertheiner, Fabio Silvio Taccone, Matteo Di Nardo, Lars Mikael Broman, Roberto Lorusso

**Affiliations:** 1 2nd Intensive Care Unit, UOC Anestesia e Rianimazione II Cardiopolmonare, Fondazione IRCCS Policlinico San Matteo, 27100 Pavia, Italy; m.belliato@gmail.com; 2 Department of Clinical-Surgical, Diagnostic and Paediatric Sciences, Unit of Anaesthesia and Intensive Care, University of Pavia, 27100 Pavia, Italy; doc.cremascoli@gmail.com (L.C.); magiqu@gmail.com (M.G.Q.); 3 1st Intensive Care Unit, UOC Anestesia e Rianimazione I, Fondazione IRCCS Policlinico San Matteo, 27100 Pavia, Italy; fioreferrari28@gmail.com; 4 International Renal Research Institute of Vicenza (IRRIV) and Department of Nephrology, Dialysis and Transplantation, 36100 Vicenza, Italy; 5 Department of Internal Medicine II, Cardiology and Pneumology, Intensive Care, University Hospital Regensburg, 93053 Regensburg, Germany; christoph.fisser@ukr.de (C.F.); maxmalfertheiner@gmail.com (M.V.M.); 6 Department of Intensive Care, Erasme Hospital, Université Libre de Bruxelles, Cliniques Universitaires de Brussels, 1070 Brussels, Belgium; ftaccone@ulb.ac.be; 7 Pediatric Intensive Care, Bambino Gesù Children’s Hospital, IRCCS, 00165 Rome, Italy; matteo.dinardo@opbg.net; 8 ECMO Centre Karolinska, Astrid Lindgren Children’s Hospital, Karolinska University Hospital, and Department of Physiology and Pharmacology, Karolinska Institutet, 171 64 Solna (Stockholm), Sweden; lars.broman@sll.se; 9 Department of Cardio-Thoracic Surgery, Heart and Vascular Centre, Maastricht University Medical Centre (MUMC), 6229 HX Maastricht, The Netherlands; roberto.lorussobs@gmail.com; 10 Cardiovascular Research Institute Maastricht (CARIM), 6229 ER Maastricht, The Netherlands

**Keywords:** veno-venous extracorporeal membrane oxygenation, acute respiratory distress syndrome, ventilator-induced lung injury, mechanical ventilation, mechanical power, respiratory rate

## Abstract

Mechanical power (MP) represents a useful parameter to describe and quantify the forces applied to the lungs during mechanical ventilation (MV). In this multi-center, prospective, observational study, we analyzed MP variations following MV adjustments after veno-venous extra-corporeal membrane oxygenation (VV ECMO) initiation. We also investigated whether the MV parameters (including MP) in the early phases of VV ECMO run may be related to the intensive care unit (ICU) mortality. Thirty-five patients with severe acute respiratory distress syndrome were prospectively enrolled and analyzed. After VV ECMO initiation, we observed a significant decrease in median MP (32.4 vs. 8.2 J/min, *p* < 0.001), plateau pressure (27 vs. 21 cmH_2_O, *p* = 0.012), driving pressure (11 vs. 8 cmH_2_O, *p* = 0.014), respiratory rate (RR, 22 vs. 14 breaths/min, *p* < 0.001), and tidal volume adjusted to patient ideal body weight (V_T_/IBW, 5.5 vs. 4.0 mL/kg, *p* = 0.001) values. During the early phase of ECMO run, RR (17 vs. 13 breaths/min, *p* = 0.003) was significantly higher, while positive end-expiratory pressure (10 vs. 14 cmH_2_O, *p* = 0.048) and V_T_/IBW (3.0 vs. 4.0 mL/kg, *p* = 0.028) were lower in ICU non-survivors, when compared to the survivors. The observed decrease in MP after ECMO initiation did not influence ICU outcome. Waiting for large studies assessing the role of these parameters in VV ECMO patients, RR and MP monitoring should not be underrated during ECMO.

## 1. Introduction

The complex interaction between mechanical ventilation (MV) and the native lung may promote ventilator-induced lung injury (VILI), especially in patients suffering from the acute respiratory distress syndrome (ARDS) [1], which would lead to gas exchange impairment and decreased respiratory system compliance [2]. The concept of lung protective ventilation has been developed and proven to reduce mortality in ARDS patients [3]. Low tidal volume (V_T_), low driving pressure (ΔP) [4,5], and high positive end-expiratory pressure (PEEP) are recommended to minimize the forces applied to the lungs and to avoid cyclic collapse and reopening of alveoli [6].

Nowadays, extracorporeal membrane oxygenation (ECMO) has become an effective and safe intervention in severe ARDS, with an increasing use in clinical practice [7,8]. In the absence of any standardized protocol, strategies to mitigate VILI during ECMO [9] rely on expert opinions [10]; nevertheless, it is generally accepted that reduced VILI would enhance lung recovery also in these patients [11,12].

To perform lung protective ventilation, it is necessary to understand how to reduce the forces applied by MV on lung tissue [13]. Stress and strain are difficult to measure in daily practice. As such, an available parameter which accounts for most of the potential causes of VILI has been recently introduced [14,15]: the so-called “mechanical power” (MP) represents the total energy delivered within a given time frame to the respiratory system, expressed in joules/minute (J/min) [16]. MP is the sum of the forces acting on the lung surface during MV, which are, according to the equation of motion: respiratory rate (RR), V_T_, respiratory system elastance, inspiratory-to-expiratory time ratio, airway resistance, and PEEP [14,16]. MP has been suggested as a main determinant of VILI pathogenesis [17,18,19]. Additionally, it was independently associated with intensive care unit (ICU) mortality, ICU and hospital length of stay, and ventilator-free days in ARDS patients, even when low V_T_ and low ΔP were applied [20]. 

In this study, we aimed to assess the changes in MV parameters after the initiation of VV ECMO in a cohort of ARDS patients. The primary aim of this pilot study was to describe and quantify the variation of MP resulting from the adjustment of MV settings. The second aim was to evaluate whether MP was associated with ICU mortality when analyzed during the initial phases of ECMO run. 

## 2. Materials and Methods 

### 2.1. Study Population

This multi-center, prospective, observational study was performed between December 2015 and June 2017 at three European ICUs experienced with ECMO: Foundation IRCCS San Matteo Hospital (Pavia, Italy), Universitätsklinikum Regensburg (Regensburg, Germany) and Hôpital Erasme (Brussels, Belgium). Institutional review boards of each center approved the study protocol. Eligible candidates were screened from local investigators. Informed consent was acquired retrospectively from every patient or a member of the family, as appropriate [21], according to local laws.

Patients enrolled in this study had severe ARDS according to the Berlin Definition [22], unresponsive to maximal medical therapy. VV ECMO was employed according to Extracorporeal Life Support Organization (ELSO) guidelines for adult respiratory failure [23]. Exclusion criteria were age < 18 years, mechanical ventilation ≥ 7 days before ECMO implementation, futility, extracorporeal support as bridge to lung transplantation and involvement in other interventional trials conflicting with the present study. Drop-out criteria were patient extubation during ECMO, relocation to another ICU, unpredicted situations that would not allow detailed evaluation and continuous monitoring (i.e., failing circuits). Patients study recruitment was completed within 12 h from ECMO initiation. Standard care was provided according to clinical practice.

### 2.2. Data Collection

We registered all data in an anonymous Excel file (Excel 2010, v14.0. *Microsoft Corporation*, Redmond, WA, USA). After study enrolment, patient demographic and anthropometric data, as well as diagnosis upon hospital and ICU admission, were collected. Sequential Organ Failure Assessment (SOFA) score [24] and Simplified Acute Physiology Score (SAPS) II [25] on ICU admission and Respiratory ECMO Survival Prediction RESP (RESP) score [26] before ECMO implementation were also recorded.

MV settings [PEEP (cmH_2_O), plateau pressure (P_plat_, cmH_2_O), peak respiratory pressure (P_peak_, cmH_2_O), ΔP (cmH_2_O), RR (breaths/min), V_T_ adjusted to patient ideal body weight (V_T_/IBW, mL), inspired oxygen fraction (F_i_O_2_, ratio)] were recorded simultaneously, the first time within 12 h from the initiation of VV ECMO, then once a day during the entire ECMO length. Last reported MV settings before ECMO cannulation were also collected. P_peak_ was considered equal to P_plat_ in pressure controlled MV modes. ΔP was directly computed as the difference between the reported values of P_plat_ and PEEP [4]. V_T_/IBW was calculated according to the Devine formula [27]. VV ECMO run and ICU stay lengths were recorded, such as ECMO successful weaning and ICU mortality.

Each MP record was calculated retrospectively from MV data, using an energy calculator, developed by Gattinoni et al. for this specific purpose [28]. We adopted a simplified formula derived from the extended equation [14], as follows:

(1) $$Power_{rs}=0.098\times RR\times V_{T}\times \left(P_{peak}-\frac{1}{2}\Delta P\right)$$

MP values were obtained by filling in the software *RR*, *V_T_* and *P_peak_*. This mathematical simplification of the original mechanical power formula allows an easier computation of MP at bedside. As stated in the original paper [14], this formula is limited, as the extended one, by the assumption of a linear compliance of the respiratory system in the range of considered pressures and volumes.

### 2.3. Statistical Analysis

Statistical analysis was conducted with STATA [Stata Statistical Software: Release 14 (2015). *StataCorp LP*, College Station, TX, USA] and significance level was set at 0.05. Categorical data are expressed as counts and percentage; continuous data are presented as median (IQR, 25th-75th percentiles). 

Study patients were evaluated according to the ICU outcome (i.e., non-survivors vs. survivors). Baseline and clinical characteristics of the patients were compared among these groups using Fisher’s exact test and Mann Whitney U-test, as appropriate. To estimate the MV variations after ECMO, the mean values of the first 48 h of ECMO run was considered for each patient. Wilcoxon rank-sum test for paired data was used to test changes in MV parameters before and after ECMO initiation. 

The duration of ECMO therapy was divided into quartiles for each patient and the MV variables mean values of the first quartile were considered. As such, MV parameters were analyzed according to the ICU outcome, using the Wilcoxon rank-sum test. Considering the first quartile of ECMO run, MV continuous variables were categorized according to the mean values of our sample. MP value was therefore categorized according to the threshold for risk of VILI from an experimental model (i.e., 12 J/min) [17]. Pearson’s chi-square test was then used to analyze the correlation between MV parameters (including MP) and ICU mortality. 

## 3. Results

### 3.1. Study Population

From a total of 151 patients undergoing VV ECMO during the study period, 35 patients were included in the final analysis. The most frequent diagnosis was primary ARDS from bacterial (n = 20, 57%) or viral pneumonia (n = 6, 17%). Other common diagnoses were secondary ARDS from abdominal sepsis (n = 3, 9%) and major trauma (n = 2, 6%). Among the remaining patients (n = 4, 11%), three developed a primary ARDS after fungal pneumonia, lung transplantation and chemotherapy, respectively, while the fourth patient developed ARDS secondary to major surgery. 

Median age of the study cohort was 53 (40–64) years; other demographic and anthropometric baseline data among the study population are listed in Table 1. Prognostic scores on ICU admission and the mechanical ventilation settings before ECMO initiation are also reported in Table 1. Median length of ICU stay was 20 (11–33) days, while median duration of ECMO run was 10 (4–15) days. ECMO weaning was successful in 28 patients (80%); the remaining seven patients died while on ECMO support. Four additional patients died due to complications during the ICU stay after successful ECMO removal; as such, overall ICU mortality was 32% (11/35 patients). 

Table 1 reports the main differences between ICU non-survivors and survivors. SAPS II score [68 (51–80) vs. 49 (37–60), *p* = 0.005] and PEEP before ECMO [10 (8–12) vs. 15 (12–16), *p* = 0.03] were significantly different between these groups; ICU stay [11 (5–15) vs. 28 (16–38) days, *p* = 0.009] and duration of ECMO run [4 (2–11) vs. 10 (5–16) days, *p* = 0.031) were notably shorter in ICU non-survivors when compared to others.

### 3.2. MV Parameters before and after ECMO Initiation

**Figure 1:**
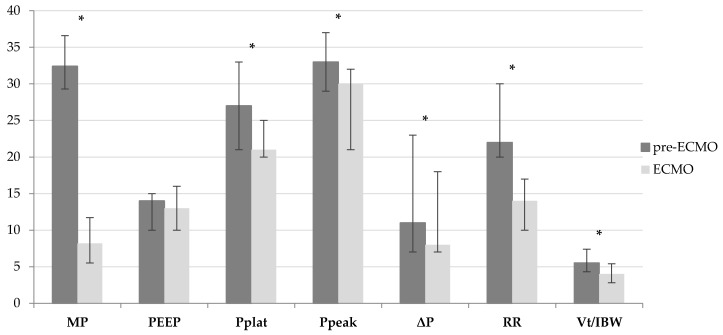
Differences in MV parameters before (pre-ECMO) and during the first 48 h after VV ECMO initiation (ECMO). [MV mechanical ventilation, VV ECMO veno-venous extracorporeal membrane oxygenation, MP mechanical power, PEEP positive end-expiratory pressure, P_plat_ plateau pressure, P_peak_ peak pressure, ΔP driving pressure, RR respiratory rate, V_T_/IBW patient ideal body weight adjusted tidal volume].

Table 2 and Figure 1 show main differences in MV parameters before and after VV ECMO initiation. In particular, a significant reduction in MP [32.4 (29.3–36.6) vs. 8.2 (5.5–11.7) J/min, *p* < 0.001] was observed. Similarly P_plat_ [27 (21–33) vs. 21 (20–25) cmH_2_O, *p* = 0.012], P_peak_ [33 (29–37) vs. 30 (21–32) cmH_2_O, *p* < 0.001], ΔP [11 (7–23) vs. 8 (7–10) cmH_2_O, *p* = 0.014], RR [22 (20–30) vs. 14 (10–17) breaths/min, *p* < 0.001], V_T_/IBW [5.5 (4.3–7.4) vs. 4.0 (2.8–5.4 mL/kg, *p* = 0.001] and F_i_O_2_ [1.0 (0.80–1.00) vs. 0.60 (0.40–0.80), *p* < 0.001] significantly decreased after ECMO initiation.

### 3.3. MV Parameters during Early Phases of ECMO Run and ICU Mortality

As shown in Table 3, during the first quartile of ECMO run, RR [17 (15–25) vs. 13 (10–16) breaths/min, *p* = 0.003] was significantly higher in ICU non-survivors than survivors. Similarly, PEEP [10 (8–12) vs. 14 (11–16) cmH_2_O, *p* = 0.048] and V_T_/IBW [3.0 (2.0–4.0) vs. 4.0 (3.5–6.0) mL/kg, *p* = 0.028] were significantly lower in ICU non-survivors. Moreover, a RR greater than 15 breaths/min correlated to an increase in ICU mortality (*p* = 0.008, Table 4). No further differences in other MV variables values (including MP) were detected between these groups.

## 4. Discussion

In this pilot study, we evaluated MP and other MV parameters after VV ECMO initiation and its prognostic role in a selected ARDS population. We observed that an ultra-protective lung ventilation strategy was applied in these patients, with a consequent significant reduction in MP after ECMO initiation. Nevertheless, RR, PEEP, and V_T_/IBW, but not MP, were the MV variables that differed during the early phases of ECMO run between ICU non-survivors and survivors.

During ARDS, MV is grounded on minimizing V_T_, with a reduction in ΔP, and maintaining adequate level of PEEP [6]. Nowadays, it is possible to safely rely on VV ECMO support for both oxygenation and carbon dioxide clearance, limiting VILI and allowing the lungs to recover [7,12]. Despite lung-protective ventilation being relatively standardized in ARDS patients without ECMO [29], there is no specific recommendation on how to manage native lung ventilation and respiratory workload during VV ECMO [30,31], which resulted into different strategies in clinical practice [3,32,33]. As for all ARDS patients, ΔP has been shown to be an important MV variable correlating with mortality in VV ECMO [34,35,36]; however, there are no large studies showing the effects of ΔP-individualized MV therapy in ECMO patients and lung recovery or patients’ survival [37].

In the last years, the severity of VILI has been related to the MP, which represents the amount of energy transmitted during MV to the respiratory system per time unit [14]. Taking into account some potential limitations [15,38,39], MP might represent a useful tool to optimize MV and potentially limit VILI [16,17,18,19] during ECMO. In our multi-center observational study, the median pre-ECMO MP value of 32.4 J/min was particularly high, considering the threshold (i.e., 17.0 J/min), which has been associated with an increased risk of mortality [20]. This is an interesting finding, since it reported the inadequacy of MV settings in severe ARDS patients failing to respond to conventional therapies [3,4]. After MV adjustments following ECMO initiation, the median MP value dropped significantly to 8.2 J/min, which is below the reported threshold (i.e., 12 J/min) which was associated with an increased risk of VILI in experimental models [17]. This reduction in MP is consistent with results from an international study from experienced ECMO centers [37]. Taking into account MV settings, our results showed that ultra-protective ventilation strategy [32], with significant reductions in V_T_/IBW (from 5.5 to 4.0 mL/kg), P_plat_ (from 27 to 21 cmH_2_O), ΔP (from 11 to 8 cmH_2_O) and RR (from 22 to 14 breaths/min) was feasible in all patients after ECMO initiation. However, lung ventilation parameters were also within the “protective” ranges before ECMO initiation [3], with levels of PEEP (from 14 to 13 cmH_2_O) indicating the maintenance of an “open lung” strategy [40,41], even during ECMO. These findings suggest the importance of MP monitoring at the bedside, as lung stress may occur even within acceptable ranges of V_T_, P_plat_, and PEEP and might prompt an earlier use of extra-corporeal therapies to reduce the occurrence of VILI. Whether high PEEP levels, which are relevant to avoid alveolar de-recruitment during ECMO [10,42], can also influence patients’ outcome remains to be demonstrated; also, individualized PEEP levels using MP monitoring or other techniques [43,44] remains a challenging issue that requires further investigation.

In this study, the ICU mortality (32%) was comparable to other reports [37]. According to the baseline data of our population, the higher SAPS II score in non-survivors (68 vs. 49) suggested a possible negative effect on outcomes of extra-pulmonary organ failure before VV ECMO. However, median SAPS II values were below the threshold of 80, which has been suggested as an indicator of poor outcome in ECMO patients [45,46]. This finding confirms the need for a complete evaluation of patients’ conditions before ECMO and underlines the utility of prognostic scores to identify patients who are more likely to benefit from ECMO therapy. 

The comparison of MV parameters between ICU non-survivors and survivors was limited to the first quartile of ECMO length for each patient, because this period requires the maximal effort to optimize lung protection and to limit VILI [6,47]. Later on during the ECMO run, when healing of lung parenchyma takes place, a less-protective approach is possible and efforts can be directed to promote ECMO weaning [48,49]. The lack of association between early MP changes and mortality is not inconsistent with this approach. Mortality during ECMO is not only due to persistent lung injury but also determined by secondary complications (i.e., bleeding, sepsis, acute ischemic stroke) and decisions to withdraw life-sustaining therapies. Nonetheless, MP should not be overlooked while on ECMO. Other MV parameters that account for MP computation, such as PEEP, V_T_/IBW, and RR should also be carefully monitored. The lower PEEP values in ICU non-survivors (10 vs. 14 cmH_2_O) could support the open lung strategy [40,41,42,43]. At the same time, the lower V_T_/IBW in ICU deaths (3.0 vs. 4.0 mL/kg) might suggest a limit value in V_T_ reduction, even on ECMO [9,50,51]. We also observed differences in ICU mortality when RR was categorized according to a specific threshold (i.e., 15 breaths/min). In clinical studies, RR has progressively received more attention, gaining relevance over V_T_ and airways pressures on VILI prevention and limitation [52]. A near-apneic MV (RR 5 breaths/min) revealed decreased lung injury in ARDS patients treated with VV ECMO [53]. Furthermore, while maintaining other MV parameters stable (P_plat_, ΔP, V_T_, PEEP), an animal model showed for each 5-fold increase in RR an 11-fold increase in MP [17]. To date, the literature is still lacking in well-defined clinical recommendations concerning RR for MV adjustment during VV ECMO [23,54,55]. Our results corroborate the crucial role of the duration of exposure to the delivered injuring strain (i.e., numbers of cycles) to determine thresholds [56], highlighting RR as one of the main determinants for energy transmission [18,30]. 

### Study Limitations

This pilot study has several limitations, mostly due to the small sample size. As a consequence, we had a limited number of observations to run a multivariable model to assess independent predictors of ICU mortality. We involved three different experienced ECMO centers, with specific patient selection criteria, which could reduce generalizability of overall findings. For practical issues, MV parameters and settings were only collected once a day and may thus not properly describe all the potential settings changes in a 24-h period, especially in the first stages of ECMO run. For this reason, we evaluated the MV variations after ECMO initiation taking into account, for each patient, the mean values of the first 48 h of ECMO length. Furthermore, our population showed a significant variability among ECMO run duration, with a mean value of 10 days and a standard deviation of 8 days. We believe that the correlation analysis between MV settings and ICU mortality based on the first quartiles of ECMO length for each patient, rather than on a fixed number of days (as presented in other VV ECMO studies), better mirrors the actual acute phase of ARDS for a given patient. Lastly, we would underline that all the MP values were calculated from the MV parameters’ datasheet. 

## 5. Conclusions

There are no recommendations on optimal MV settings during VV ECMO. The results from this pilot study confirmed that VV ECMO allows a significant reduction of MP. Early MP values did not predict patients’ outcome in this cohort. Further larger studies are needed to assess the prognostic role of MP and other MV parameters in VV ECMO patients. Importantly, these parameters should still be adequately monitored in this setting. 

## Figures and Tables

**Table 1:** Study population baseline demographic and anthropometric characteristics, ICU admission prognostic scores, MV settings before VV ECMO initiation (pre-ECMO), ICU stay and ECMO run length. Variables comparison according to ICU mortality. [ICU intensive care unit, MV mechanical ventilation, VV ECMO veno-venous extracorporeal membrane oxygenation, BMI body mass index, SOFA sequential organ failure assessment, SAPS simplified acute physiology score, RESP respiratory ECMO survival prediction, MP mechanical power, PEEP positive end-expiratory pressure, P_plat_ plateau pressure, P_peak_ peak pressure, ΔP driving pressure, RR respiratory rate, V_T_/IBW patient ideal body weight adjusted tidal volume, F_i_O_2_ lung inspiratory oxygen fraction].

	Study Population (n = 35)	ICU Non-Survivors (n = 11)	ICU Survivors (n = 24)	*p*-Value
**Age** [years; median value (25p–75p)]	**53** (40–64)	**53** (39–67)	**53** (42–62)	0.902
**Male sex** [n; %]	**24** (68)	**6** (54)	**18** (75)	0.233
**Weight** [kg; median value (25p–75p)]	**84** (70–110)	**75** (60–85)	**85** (77–118)	0.057
**Height** [cm; median value (25p–75p)]	**175** (169–180)	**171** (167–177)	**177** (169–180)	0.182
**BMI** [kg/m^2^; median value (25p–75p)]	**27** (24–35)	**24** (21–29)	**28** (26–37)	0.145
**SOFA score** [n; median value (25p–75p)]	**12** (9–17)	**14** (11–18)	**12** (8–17)	0.228
**SAPS II score** [n; median value (25p–75p)]	**53** (42–68)	**68** (51–80)	**49** (37–60)	0.005
**RESP score** [n; median value (25p–75p)]	**−4** (−7–0)	**−6** (−9–−1)	**−3** (−7–0)	0.398
**pre-ECMO MP** [J/min; median value (25p–75p)]	**32.4** (29.3–36.6)	**31.1** (29.4–35.8)	**32.6** (23.6–38.2)	0.918
**pre-ECMO PEEP** [cmH_2_O; median value (25p–75p)]	**14** (10–15)	**10** (8–12)	**15** (12–16)	0.032
**pre-ECMO P_plat_** [cmH_2_O; median value (25p–75p)]	**27** (21–33)	**30** (19–38)	**27** (21–31)	0.506
**pre-ECMO P_peak_** [cmH_2_O; median value (25p–75p)]	**33** (29–37)	**34** (30–38)	**33** (28–35)	0.604
**pre-ECMO ΔP** [cmH_2_O; median value (25p–75p)]	**11** (7–23)	**22** (7–29)	**13** (7–13)	0.237
**pre-ECMO RR** [breaths/min; median value (25p–75p)]	**22** (20–30)	**25** (23–40)	**21** (18–27)	0.059
**pre-ECMO V_T_/IBW** [mL/kg; median value (25p–75p)]	**5.5** (4.3–7.4)	**5.1** (4.1–6.9)	**5.9** (4.8–7.4)	0.441
**pre-ECMO F_i_O_2_** [ratio; median value (25p–75p)]	**1.00** (0.80–1.00)	**1.00** (0.80–1.00)	**1.00** (0.80–1.00)	0.929
**ICU stay length** [days; median value (25p–75p)]	**20** (11–33)	**11** (5–15)	**28** (16–38)	0.009
**VV ECMO run length** [days; median value (25p–75p)]	**10** (4–15)	**4** (2–11)	**10** (5–16)	0.031

**Table 2:** Differences in MV parameters before (pre-ECMO) and during the first 48 h after VV ECMO initiation (ECMO). [MV mechanical ventilation, VV ECMO veno-venous extracorporeal membrane oxygenation, Δ% percentage variation, MP mechanical power, PEEP positive end-expiratory pressure, P_plat_ plateau pressure, P_peak_ peak pressure, ΔP driving pressure, RR respiratory rate, V_T_/IBW patient ideal body weight adjusted tidal volume, F_i_O_2_ lung inspiratory oxygen fraction].

	pre-ECMO (n = 35)	ECMO (n = 35)	Δ%	*p*-Value
**MP** [J/min; median value (25p–75p)]	**32.4** (29.3–36.6)	**8.2** (5.5–11.7)	−74.7%	<0.001
**PEEP** [cmH_2_O; median value (25p–75p)]	**14** (10–15)	**13** (10–16)	−7.1%	0.390
**P_plat_** [cmH_2_O; median value (25p–75p)]	**27** (21–33)	**21** (20–25)	−22.2%	0.012
**P_peak_** [cmH_2_O; median value (25p–75p)]	**33** (29–37)	**30** (21–32)	−9.1%	<0.001
**ΔP** [cmH_2_O; median value (25p–75p)]	**11** (7–23)	**8** (7–10)	−27.3%	0.014
**RR** [breaths/min; median value (25p–75p)]	**22** (20–30)	**14** (10–17)	−36.4%	<0.001
**V_T_/IBW** [mL/kg; median value (25p–75p)]	**5.5** (4.3–7.4)	**4.0** (2.8–5.4)	−27.3%	0.001
**F_i_O_2_** [ratio; median value (25p–75p)]	**1.00** (0.80–1.00)	**0.60** (0.40–0.80)	−40.0%	<0.001

**Table 3:** Comparison of MV parameters during the first quartile of VV ECMO length, according to the ICU mortality. [MV mechanical ventilation, VV ECMO veno-venous extracorporeal membrane oxygenation, ICU intensive care unit, MP mechanical power, PEEP positive end-expiratory pressure, P_plat_ plateau pressure, P_peak_ peak pressure, ΔP driving pressure, RR respiratory rate, V_T_/IBW patient ideal body weight adjusted tidal volume, F_i_O_2_ lung inspiratory oxygen fraction].

	Study Population (n = 35)	ICU Non-Survivors (n = 11)	ICU Survivors (n = 24)	*p*-Value
**MP** [J/min; median value (25p–75p)]	**8.0** (5.0–14.0)	**8.0** (5.0–20.0)	**8.0** (6.0–13.0)	0.530
**PEEP** [cmH_2_O; median value (25p–75p)]	**12** (10–16)	**10** (8–12)	**14** (11–16)	0.048
**P_plat_** [cmH_2_O; median value (25p–75p)]	**22** (20–25)	**22** (19–29)	**22** (20–25)	0.880
**P_peak_** [cmH_2_O; median value (25p–75p)]	**26** (22–31)	**30** (24–34)	**26** (21–30)	0.174
**ΔP** [cmH_2_O; median value (25p–75p)]	**10** (7–12)	**12** (7–15)	**9** (6–12)	0.229
**RR** [breaths/min; median value (25p–75p)]	**14** (10–17)	**17** (15–25)	**13** (10–16)	0.003
**V_T_/IBW** [mL/kg; median value (25p–75p)]	**4.0** (3.0–5.0)	**3.0** (2.0–4.0)	**4.0** (3.5–6.0)	0.028
**F_i_O_2_** [ratio; median value (25p–75p)]	**0.55** (0.43–0.73)	**0.43 **(0.40–0.73)	**0.57** (0.50–0.75)	0.345

**Table 4:** Association with ICU mortality, according to the categorization of MV parameters during the first quartile of VV ECMO run. [ICU intensive care unit, MV mechanical ventilation, VV ECMO veno-venous extracorporeal membrane oxygenation, MP mechanical power, PEEP positive end-expiratory pressure, P_plat_ plateau pressure, P_peak_ peak pressure, ΔP driving pressure, RR respiratory rate, V_T_/IBW patient ideal body weight adjusted tidal volume, F_i_O_2_ lung inspiratory oxygen fraction].

	Variable Categorization	Pearson’s Chi-Square Test	*p*-Value
**MP** (J/min)	≤12; >12	0.475	0.491
**PEEP** (cmH_2_O)	<12; ≥12	2.828	0.093
**P_plat_** (cmH_2_O)	≤23; >23	2.198	0.138
**P_peak_** (cmH_2_O)	≤27; >27	0.957	0.328
**ΔP** (cmH_2_O)	<10; ≥10	1.247	0.264
**RR** (breaths/min)	<15; ≥15	7.098	0.008
**V_T_/IBW** (mL/kg)	<4.5; ≥4.5	1.847	0.174
**F_i_O_2_** [ratio]	≤0.6; >0.6	0.088	0.766
